# Conditional Transgenic Expression of PIM1 Kinase in Prostate Induces Inflammation-Dependent Neoplasia

**DOI:** 10.1371/journal.pone.0060277

**Published:** 2013-04-02

**Authors:** Maja Narlik-Grassow, Carmen Blanco-Aparicio, Yolanda Cecilia, Marco Perez, Sandra Muñoz-Galvan, Marta Cañamero, Amancio Carnero

**Affiliations:** 1 Experimental Therapeutics programme, Spanish National Cancer Research Centre, Madrid, Spain; 2 Biotechnology programme, Spanish National Cancer Research Centre, Madrid, Spain; 3 Instituto de Biomedicina de Sevilla (IBiS), Hospital Universitario Virgen del Rocio, Consejo Superior de Investigaciones Cientificas, Universidad de Sevilla, Sevilla, Spain; Thomas Jefferson University, United States of America

## Abstract

The Pim proteins are a family of highly homologous protein serine/threonine kinases that have been found to be overexpressed in cancer. Elevated levels of Pim1 kinase were first discovered in human leukemia and lymphomas. However, more recently Pim1 was found to be increased in solid tumors, including pancreatic and prostate cancers, and has been proposed as a prognostic marker. Although the Pim kinases have been identified as oncogenes in transgenic models, they have weak transforming abilities on their own. However, they have been shown to greatly enhance the ability of other genes or chemical carcinogens to induce tumors. To explore the role of Pim1 in prostate cancer, we generated conditional Pim1 transgenic mice, expressed Pim1 in prostate epithelium, and analyzed the contribution of PIM1 to neoplastic initiation and progression. Accordingly, we explored the effect of PIM1 overexpression in 3 different settings: upon hormone treatment, during aging, and in combination with the absence of one *Pten* allele. We have found that Pim1 overexpression increased the severity of mouse prostate intraepithelial neoplasias (mPIN) moderately in all three settings. Furthermore, Pim1 overexpression, in combination with the hormone treatment, increased inflammation surrounding target tissues leading to pyelonephritis in transgenic animals. Analysis of senescence induced in these prostatic lesions showed that the lesions induced in the presence of inflammation exhibited different behavior than those induced in the absence of inflammation. While high grade prostate preneoplastic lesions, mPIN grades III and IV, in the presence of inflammation did not show any senescence markers and demonstrated high levels of Ki67 staining, untreated animals without inflammation showed senescence markers and had low levels of Ki67 staining in similar high grade lesions. Our data suggest that Pim1 might contribute to progression rather than initiation in prostate neoplasia.

## Introduction

The Pim proteins are a family of short-lived, serine/threonine kinases that are highly conserved throughout evolution in multicellular organisms. This family of kinases is composed of three different members (*Pim1*, *Pim2* and *Pim3*) that are highly homologous at the amino acid level [1], yet they differ in their tissue distributions [2]. Functional redundancy of the three Pim kinases has been shown *in vitro* [3], [4] and *in vivo* [5], [6].

Pim kinases are primary response genes whose transcription is rapidly upregulated upon mitogenic stimuli and are transiently induced in response to a wide range of growth factors [7], [8], [9], including IL-2, IL-3, GM-CSF and IFN-γ. The majority of these factors transduce their primary signal through the JAK/STAT pathway, indicating that this cascade is instrumental in regulating the expression of the *Pim* genes [5]. Additionally Pim1 is able to negatively regulate the JAK/STAT pathway by binding to SOCS proteins [10]. Gene expression of any of the 3 Pim kinases is also induced by activation of the NF-κB signaling pathway, hypoxia in solid tumors independently of HIF1α [11] and upon DNA damage by Krüppel-like factor 5 (KFL5), thereby protecting cells from apoptosis [12].

Pim kinases are not regulated by posttranslational modifications like other kinases but are primarily regulated by transcription, translation, and proteosomal degradation [13], [14], [15], [16].

Although the Pim kinases have been identified as oncogenes in transgenic models, they are only weakly transforming by themselves. However, they have been shown to greatly enhance the ability of c-myc to induce lymphomas and prostate cancer [17], [18], [19], [20], perhaps by counteracting Myc-induced apoptosis [21].

Pim kinases mediate their physiological activities through the phosphorylation of a wide range of cellular substrates such as SOCS-1 [22], [23], runt-related transcription factor 1, RuNX1, and RuNX3 [24], cell cycle regulators (such as p21^waf1^ and p27^kip1^ [25], [26], cdc25A [27] and cTAK/MARK3/Par1A), pro-apoptotic proteins (such as Bad and ASK1 [28], [29]), and transcriptional regulators (such as HP1, NFATc1, c-Myb or p100 [30], [31], [32], [33], [34]). More recently, Pim2 has been shown to phosphorylate the ribosomal protein 4E-BP1, causing its dissociation from eIF-4E and possibly affecting protein synthesis because eIF-4E is a rate-limiting factor [35]. Interestingly, several of the above-mentioned substrates are shared with the AKT kinases [21], [36], [37].

Elevated levels of Pim1 kinase were first reported in human leukemia and lymphomas [8], [38], [39]. Recently, Pim1 was found to be increased in solid tumors, including pancreatic and prostate cancers, squamous cell carcinoma, gastric, colorectal and liver carcinomas [40], [41], and liposarcoma [42]. Increased levels of Pim2 kinase have been detected in various lymphomas as well as in prostate cancer [43]. Pim3 kinase has been found to be aberrantly expressed in malignant lesions of endoderm-derived organs, the liver and pancreas, and also in Ewing's sarcoma [1].

Prostate cancer (PC) is the most common malignancy in men in the western world. PC usually develops slowly through a series of defined states, such as prostate intraepithelial neoplasia (PIN), prostate cancer *in situ*, invasive cancer and finally metastatic cancer [44]. PIN, generally known as a likely precursor for human prostate cancer, can be defined as either low grade or high grade. The latter is believed to be a precursor of prostate adenocarcinoma [45]. However, not all PIN lesions progress to invasive prostate cancer. The molecular pathways that contribute to the progression from high grade PIN lesions to prostate adenocarcinoma are still widely undetermined. Pim1 has been shown to be overexpressed in high grade PIN, which might be a sign that Pim kinases are involved in the early development of prostate malignancy [46], [47]. However, the expression of Pim1 in human prostate cell lines representing different stages of the disease demonstrated that Pim1 overexpression alone was not sufficient to transform the benign RWPEI cells to malignancy, but Pim1 did enhance the tumorigenic capabilities of LNCaP and Du145 cells *in vitro* [48] and PC3 prostate tumor cells *in vivo* [49]. It is possible that the p53-dependent induction of cell senescence induced by Pim1 limits Pim oncogenic effects on non-tumoral cells [50]. However, Pim1 interference was shown to induce growth inhibition and apoptosis in PC3 cells [51].

Pim1 expression is also increased by androgen ablation therapy (Poel 2008), and its expression is associated with hormone-refractory PC. Although Pim1 expression might not be sufficient to initiate expression of androgen-dependent genes, Pim1 might be involved in the progression from an androgen-dependent state to an androgen-independent state in PC. Recent studies have correlated Pim1 kinase with chemoresistance in prostate cancer cells, which is a common event in highly aggressive, hormone-refractory PCs [52], [53].

To explore the role of Pim1 in PC, we generated conditional Pim1 transgenic mice, expressed Pim1 in prostate epithelium, and analyzed the contribution of Pim1 to neoplastic initiation and progression. Accordingly, we explored the effect of Pim1 overexpression in 3 different settings: upon hormone treatment, during aging, and in combination with the absence of one *Pten* allele.

## Materials and Methods

### Maintenance of mouse colonies

All experiments with animals were performed with expressed approval from Centro Nacional de Investigaciones Oncologicas, CNIO, Ethical Committee for the Care and Health of Animals. All animals were kept in the CNIO animal facility according to the facility norms based on the Real Decreto 1201/2005 and sacrificed by CO_2_ inhalation either within a programmed procedure or as a humane endpoint when animals showed signs significant sickness. All efforts were made to minimize suffering. PSA61-CRE mice were a kind gift from J. Trapman. Pten KO mice were a kind gift of Dr H. Wu.

### Construction of the transgenic DNA

**Figure 1:**
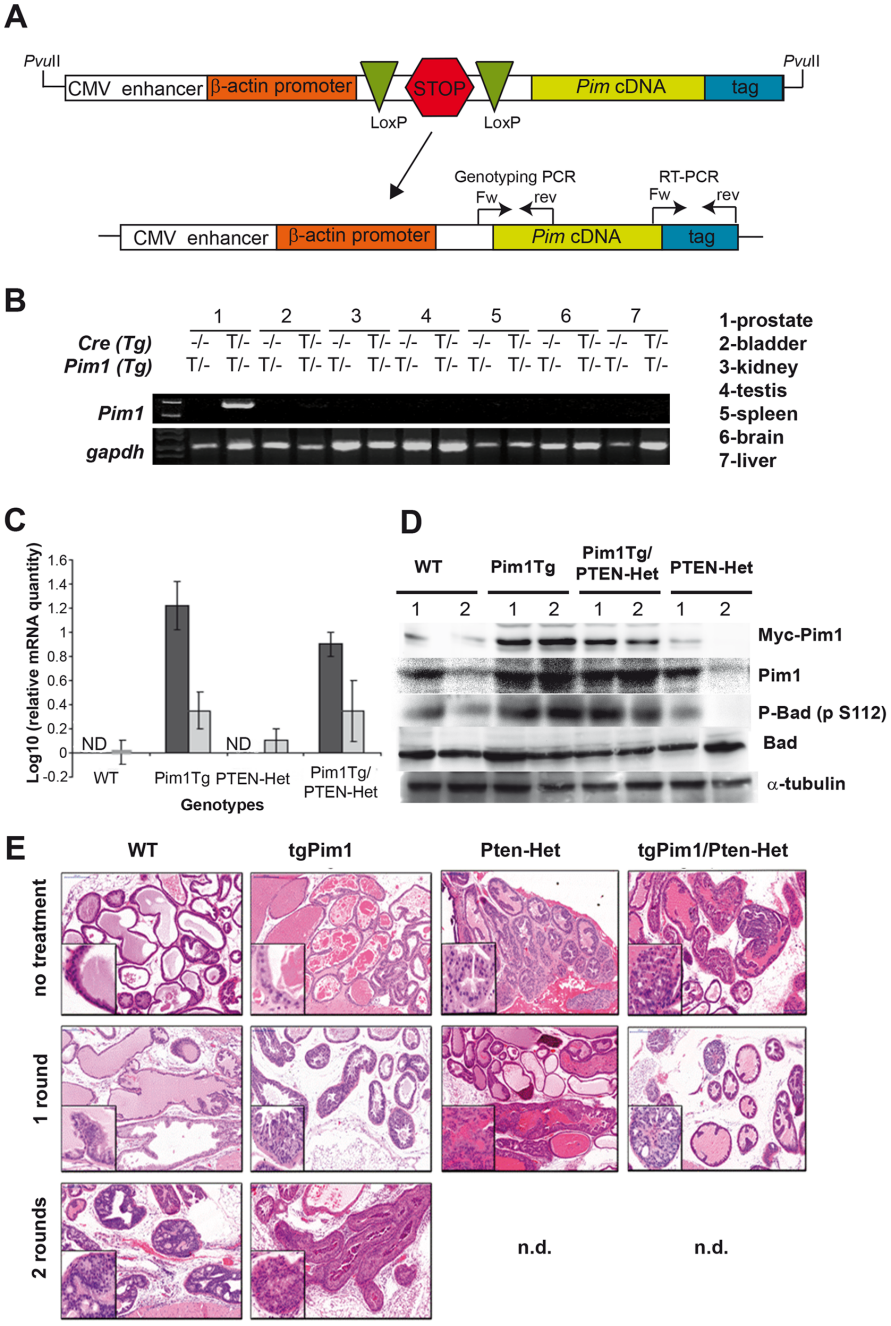
characteristics of the PIM1 conditional transgenic mice. **A**) Schematic representation of the transgene carrying Pim1. The arrow indicates the generation of tissue specific transgenic mice expressing PIM1 by crossing with mice expressing Cre-recombinase under a tissue specific promoter; the Lox/Stop/Lox cassette is excised allowing transcription. **B**) Expression of PIM1 in Pim1/PSA-Cre mice. RNA was extracted from different tissues of 10-week-old mice and the specific transgenic *Pim1* transcription analyzed by RT-PCR. **C**) Levels of human and mouse PIM1 mRNAs calculated by quantitative RT-PCR. Graph shows average (± SD) levels of expression in the prostate of human (black) or mouse (grey) PIM1 mRNA of at least two mice per genotype performed in triplicate. Data were normalized to 1 (log 10 = 0) using the levels of mouse PIM1 levels in wild type animals. ND: Not detected. Human PIM1 was not detected in WT nor PTEN-Het mice. **D**) Level of PIM1 protein in the mice of different genotypes measured by Western blot. The prostate of 2 animals (1 and 2) per cohort was processed to extract total proteins and run in a PAGE to allow further identification by western blot. Transgenic PIM1 was identified by myc-tag present in the transgene. Total PIM1 content was identified with a PIM1 antibody cross-reacting with human and mouse species. Activity was measured as Bad phosphorylation levels at S112. **E**) Representative pictures of high grade mPIN lesions developed after hormone treatment. To determine the development of mPIN lesions due to hormone treatment, 8-week-old untreated and hormone-treated (for 1 or 2 rounds) mice of each genotype were sacrificed and prostate tissue was obtained (see text for details). H&E staining of prostate tissue was used for mPIN grading. Pictures represent: No treatment: wt) pl-grade 0; tgPim1) pl-grade 1; PTEN-Het) pl-grade 5; tgPim1/PTEN-Het) pl-grade 8; 1 round of hormone treatment: wt) pl-grade 2; tgPim1) pl-grade 4; PTEN-Het) pl-grade 10; tgPim1/PTEN-Het) pl-grade 11; 2 rounds of hormone treatment: wt) pl-grade 6; tgPim1) p-grade 13.

The sequence of human *Pim1* was amplified by PCR using specifically designed primers, cDNA from human IMR90 cells as a template, and a Myc-tag. Human Pim1 then was cloned into the pVL-1 vector (see fig. 1A). The DNA construct was linearized and injected into embryonic stem cells. The embryonic stem cells were then transferred into pseudo pregnant mice. Embryonic stem cell injection, selection and transfer were carried out by the Transgenic Mice Unit of the CNIO according to their standard protocols. Mice born after embryonic stem cell injection were genotyped using specifically designed primers, crossed with wild-type (WT) C57/Bl6 mice, and the resultant pups were genotyped to verify germ line transmission. Founder mice were then bred to verify conditional transgene expression by RT-PCR. Primers designed specifically for human Pim1 kinase, which do not amplify mouse Pim genes, were used for all PCR experiments and subsequent genotyping of mice (see Fig. 1A, Table S1 for primer sequence and PCR programs).

### Genotyping of mouse lines

Four weeks after birth, the mice were weaned and 2–3 mm sections of the tails were cut, placed into 1.5 ml Eppendorf tubes, and stored at −80°C. To lyse the tail tissue, 400 µl of lysis buffer (50 mM KCl, 1.5 mM MgCl2, 10 mM Tris-HCl (pH 8.3; all from Merck), 0.45% IGEPAL CA 630 (NP40, Sigma), 0.45% Tween20 (Sigma), 10 mg/ml Proteinase K (Promega)) was added, and the tubes were incubated at 55°C over night while shaking at 220 rpm. After 16 h, the lysates were incubated at 95–100°C for 30 min to inactivate the proteinase K, the tubes were centrifuged at full speed for 10 sec, and 2 µl of the lysates were used for the genotyping PCR (Table S1).

### Carcinogenesis induced by Testosterone and Estradiol

The hormones testosterone (Sigma) and β-Estradiol (Sigma) were mixed with colorless silicone (Soudal) and dried for 48 h. Pellets were stamped out using a 5 mm biopsy punch (Stieffel) resulting in a 30 mg hormone/silicone pellet. We used male mice with an average age of 8 weeks. The mice were anesthetized using 2% isofluorane. A 5 mm incision was made on the lower back, and the pellets were inserted under the fur. The procedure was repeated after 8 weeks. The total doses of the implanted hormones are shown in Table S2. To ensure the health of the animals, the mice were monitored every 24–48 h (depending of the health status of each animal).

### Necropsy and pathological studies

Tissues for histopathologycal studies were fixed in 10% formalin for 24 h, dehydrated at different ethanol concentrations with xylol and embedded in paraffin at 65°C to obtain tissue blocks. Tissue fixation and paraffin embedding were carried out at the Comparative Pathology Unit at the CNIO.

### Grading mouse PIN (mPIN) lesions

The 8, 16 or 24 -week-old or 10 months male mice were sacrificed, and prostate tissue was procured and prepared for immunohistochemistry. Hematoxylin and eosin (H&E) staining of prostate tissue was used for mouse prostate intraepithelial neoplasia (mPIN) grading.

To grade the mPIN lesions, we used the consensus grading system established at the Bar Harbor Meeting, October 2001 (published by D. Cardiff et al.; American Journal of Pathology 2002; Cancer Res. 64; 2004), using mPIN grades from mPIN I to mPIN IV followed by carcinoma. For fine-tuning, we decided to use a numeric categorization system for prostatic epithelia lesions that subdivided the mPIN grades into prostate lesion grades (pl-grade) 0 to 13 (Table S6 and Figure S1). To discriminate between mPIN IV grade lesions and microinvasive carcinoma, we stained for smooth muscle actin (SMA) and cytokeratin 14 (CK14). In mPIN IV grade lesions, CK14 and SMA were detected by increased stained cells in number and size and haphazardly arrange of the basal epithelial cell layer of the prostate gland [54], whereas it's lost in a microinvasive carcinoma.

To differentiate mPIN IV grade lesions from carcinoma in the mice, staining for smooth muscle actin (SMA) and cytokeratin 14 (CK14) were performed for all high grade mPIN (mPIN IV and possible carcinoma) verifying the grading of the prostate lesions.

### Statistical data analysis

The computer program GraphPad Prism was used for all statistical analyses. To determine the statistical significance of the lesions in the prostate and the statistical significance of the differences in inflammation and incidence of pyelonephritis, a one-way ANOVA or a one-tailed Student's t-test were used as indicated in the figure legends.

### Analysis of transgene expression at the RNA level and Q-RTPCR

We analyzed the expression of different transcripts in tissues by reverse transcription-PCR (RT-PCR). Total RNA was isolated from tissues using TRI-Reagent (Molecular Research Center), treated with DNase (Roche), and reverse transcribed with random hexamer primers (Promega) and avian myeloblastosis virus-reverse transcriptase (Roche). The cDNA was amplified by PCR using specific primer combinations as described in Table S3. For the quantitative measurements, total RNA was purified using TRI-REAGENT (Molecular Research Center, Cincinnati Ohio). Reverse transcription was performed with 5 µg of mRNA using MMLV reverse transcriptase (Promega) and oligodT primer, according to the manufacturer's recommendations. To measure human and mouse PIM1 mRNA expression, real-time PCR was performed using an ABI 7900HT (Applied Biosystems). The reaction was carried in 96-well plates and QPCR reactions were run using Taqman Gene Expression assays (Applied Biosystems). Detection of bActin was used as internal control. Relative quantitation values were expressed as Log10 of Relative Quantity. Relative Quantity and statistical analysis for QPCR data were calculated using Applied Biosystem RQ Manager 1.2.1 software.

### Western blot analysis

Cells were washed twice with PBS and lysed by sonication in lysis-buffer (50 mM Tris-HCl pH 7.5, 1% NP-40, 2 mM Na3VO4, 150 mM NaCl, 20 mM Na4P2O7, 100 mM NaF and complete protease inhibitor cocktail (Roche Molecular Biochemicals). Samples were separated on 7.5–15% SDS-PAGE gels, transferred onto PVDF membranes (Immobilon-P, Millipore) and immunostained. The following primary antibodies were used: anti-PIM1 [EP2645Y] 1∶8000 (Abcam#ab75776), anti-Myc tag 1∶1000 (Abcam#ab9106), anti-Bad [Y208] 1∶2000 (Abcam#ab32445), anti-Bad (phospho S112) [EPR1891(2)] 1∶3000 (Abcam#ab129192), and horseradish peroxidase-labeled rabbit anti-mouse (Promega diluted 1∶5000) and goat anti-rabbit (Calbiochem 401315, diluted 1∶4000) secondary antibodies used as required. Proteins were visualized using the ECL detection system (Amersham Biosciences).

### Immunohistochemistry

For immunohistochemical analyses, prepared paraffin tissue blocks were cut into 2.5 µm sections using an automated microtome and were dyed with H&E or different antibodies (see tables). All staining were carried out at the Comparative Pathology Unit at the CNIO according to established protocols.

See Tables S4 and S5 for the antibody and conditions used.

## Results

### Generation of transgenic mice carrying the PIM1 transgene

We generated mouse lines that express the Pim1 transgene specifically in the prostate by crossing our Pim1 transgene with a transgenic line expressing Cre-recombinase under the PSA promoter (Pim1/PSA61-Cre mice) (Fig. 1A). The Lox/Stop/Lox cassette was excised by Cre-recombinase allowing transgene expression, which was tested by RT-PCR confirming specificity. We identified two founder Pim1 transgenic mice clearly expressing the Pim1 transgene under PSA-Cre and specifically in the prostate (fig. 1B). To evaluate the expression of the transgene, we measured the mRNA levels of PIM1 transgene and compared them to the levels of endogenous mouse PIM1 mRNA. PIM1 transgenic mRNA was not detected in wild type or PTEN-Het mice, but showed clear expression in PIM1 Tg and PIM1tg/PTEN-Het mice (Figure 1C). The levels of transgenic PIM1 mRNA are around ten fold higher in average than the levels of endogenous mouse PIM1 mRNA. The analysis of proteins in the different genotypes, also showed clear increased expression of the PIM1 protein in the transgenic animals, which also translate in a higher activity measured as Bad phosphorylation at S112 (Figure 1D).

### PIM1 cooperates with Pten loss in hormone-induced mPIN

Because Pim1 is regarded as a “weak” oncogene, we decided to study mPIN solely induced by Pim1 overexpression and also the effect of Pim overexpression in the physiological settings of hormone- induced mPIN and loss of one *Pten* allele. A summary of the mouse line genotypes used in this study are as follows: tgPim1 [(Pim1(Tg/+);PSA-CRE (Tg/+)], Pten-Het [(*Pten*(loxp/+);PSA-CRE (Tg/+)], tgPim1/Pten-Het [(Pim1(Tg/+); *Pten*(loxp/+);PSA-CRE (Tg/+)]. Total number of mice analyzed in each cohort appears in the Table S8.

**Figure 2:**
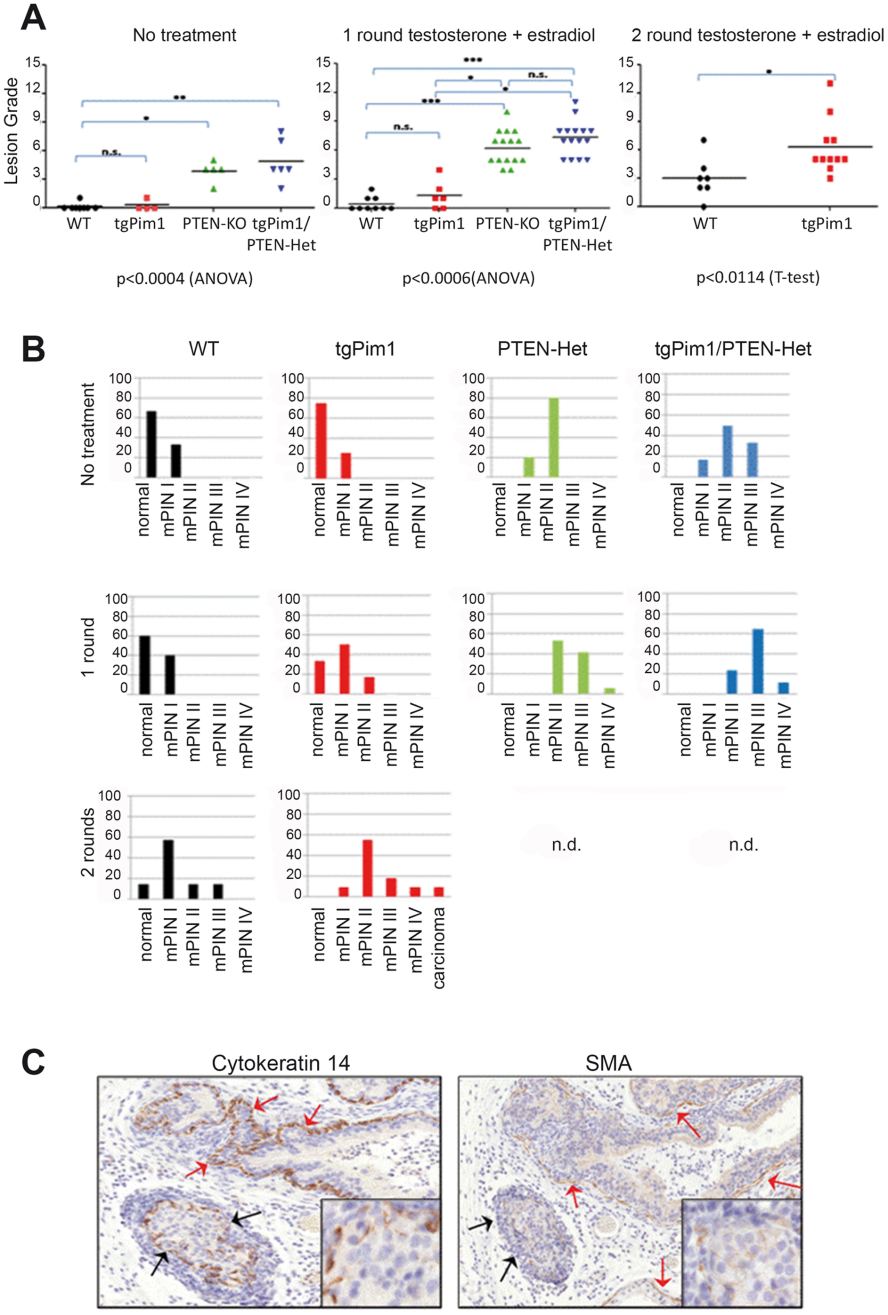
Lesions in the prostate after hormone treatment. Eight-week-old male mice of each genotype were implanted with testosterone- and estradiol – silicone pellets for 8 weeks (1 round). Wild-type mice and transgenic Pim1 mice were treated with the same pellets for another 8 weeks (2 rounds). To determine the development of prostate lesions due to hormone treatment, 8-week-old untreated mice and hormone-treated (for 1 or 2 rounds) mice of each genotype were sacrificed, and prostate tissue was obtained. **A**) Grade of prostate lesion (mPIN) reached in each treatment round. H&E staining of prostate tissue was used for prostate lesion grading, applying the Bar Harbor grading system and subdividing mPIN I-IV lesions as described in table S6. Mouse prostate lobes were procured, analyzed and the grade assigned. Graphs represent the grading observed in the different prostates analyzed; statistical relevance is also shown: * = <0.05; ** = <0.01; *** = <0.001. **B**) Incidence (in %) of mPIN lesions per genotype and round of hormone treatment. The graphs represent the percentage of mice that reach each grade in the cohort. We can observe a clear increase in the severity of the lesions achieved in mice expressing the Pim1 transgene with the different treatment rounds compared to WT mice. **C**) Differentiation of mPIN IV lesions and microinvasive carcinoma. Sixteen-week-old male mice were sacrificed, and prostate tissue was obtained and prepared for immunohistochemistry. To differentiate mPIN IV lesions (red arrows) from microinvasive carcinoma (black arrows), immunohistochemistry for cytokeratin 14 (CK14) and smooth muscle actin (SMA) was performed. To be scored as a microcarcinoma, continuous staining of both CK14 and SMA had to be absent at the basal epithelial layer of the prostate gland in question (black arrows and insert).

Before starting the hormone treatment, we assessed the lesions in untreated animals at 8 weeks of age. Whereas nearly 70% of untreated WT and tgPim1 mice showed no apparent lesions (Figure 1C), 80% of Pten-Het mice displayed type mPIN II lesions (pl-grade 4). The difference was even greater in tgPim1/Pten-Het mice, where 50% showed mPIN II lesions (pl-grade 5) and 30% reached a maximum of mPIN III (pl-grade 7) (Figure 1C). Whereas the percentage of mPIN lesion grades in WT mice after one round of hormone treatment roughly stayed the same (60% normal, 40% mPIN I), hormone treatment aggravated mPIN lesions in all other genotypes (Figures 2A and B). Over 40% of the tgPim1 mice displayed mPIN I lesions after one round of treatment, compared to 23% of the mice without treatment and 18% of the treated mice developed mPIN II lesions. The Pten-Het and tgPim1/Pten-Het mice displayed a more drastic change; both genotypes developed significantly higher mPIN grades compared to WT and tgPim1 mice after 1 round of hormone treatment (p<0.0006). After 1 round of treatment, over 50% of the Pten-Het mice exhibited mPIN II lesions (pl-grade 6), 40% showed mPIN III lesions (pl-grade 8) and one animal reached mPIN IV (pl-grade 10). The severity of the induced mPIN lesions in the tgPim1/Pten-Het mice was even greater. Over 60% of the tgPim1/Pten-Het mice showed mPIN III lesions (pl-grade 8), two animals (16%) reached mPIN IV (pl-grades 10 and 11), and 20% demonstrated only mPIN II lesions.

Although differences in mPIN grades between the Pten-Het and tgPim1/Pten-Het mice were not significant, there seemed to be an upward trend toward higher severity in the tgPim1/Pten-Het -mice, indicating a possible cooperation of PIM1 and the loss of one *Pten* allele in the severity of hormone-induced mPIN. Due to significantly increased inflammation and subsequent pyelonephritis in tgPim1/Pten-Het mice during the first round of hormone treatment, a second round of treatment was not administered and humane euthanization was performed. However, we were able to administer a second round of hormone treatment to the WT and tgPim1 mice (Fig. 2). After the second treatment round, 58% of the WT mice showed mPIN I lesions (mainly pl-grade 3), and 1 animal (13%) reached pl-grade 7. Lesions in the TgPim1 mice increased in severity significantly after the second round of hormone treatment (p<0.0114); 60% of the tgPim1 mice displayed mPIN II lesions (pl-grade 5), 20% reached mPIN III (pl-grades 6–9), and 1 animal showed mPIN IV (pl-grade 11), and 1 animal developed carcinoma (Fig. 2C).

### Pim1 does not cooperate with Pten-loss in aging-induced mPIN

**Figure 3:**
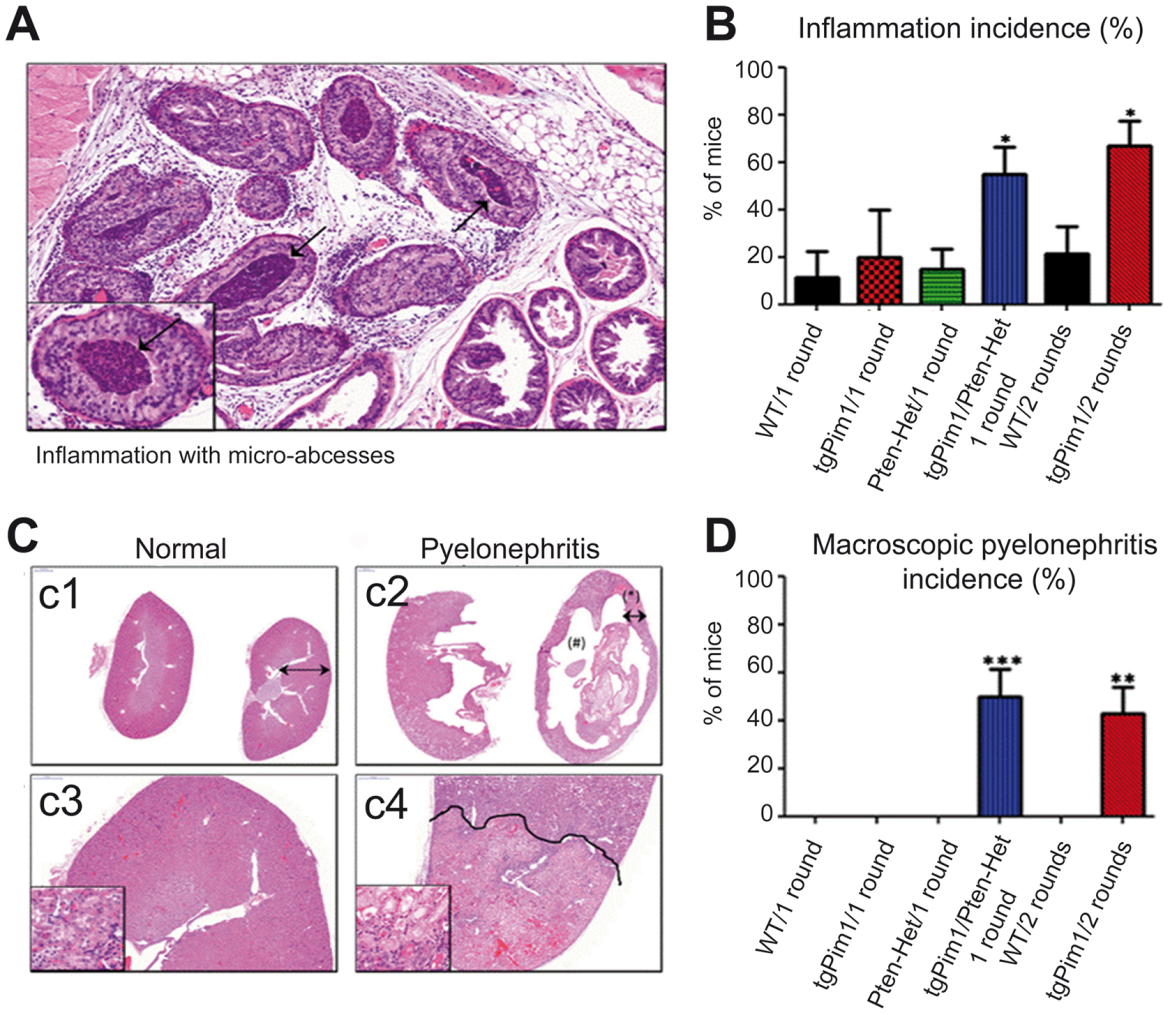
Phenotypic effect of hormone treatment. **A and B**) **Inflammation incidence in hormone- treated mice.** **A**) H&E staining of a prostate of a 16-week-old tgPim1/PTEN-Het mouse after one round of hormone treatment showing inflammation and micro-abscesses (black arrows). **B**) Percentage of inflammation incidence in every genotype after one or 2 rounds of hormone treatment, respectively. **C and D**) **Pyelonephritis incidence in hormone-treated mice.** **C**) c1 and c2: H&E staining (augmentation 0.5x, panoramic viewer) of a healthy kidney from a 24 week-old WT mouse vs. a kidney displaying pyelonephritis from a 24-week-old tgPim1 mouse, both after 2 rounds of hormone treatment. Note pelvic cystic dilatation (#) with narrowing of the remaining parenchyma (*). c3 and c4: H&E staining of the same kidneys (augmentation 25x). Note the well-demarcated areas of renal infarct in the animal with pyelonephritis, in which more than one-third of the parenchyma is affected. **D**) Percentage of pyelonephritis incidence in every genotype after one or 2 rounds of hormone treatment, respectively.

In a previous study, the loss of one Pten allele in the prostate induced high grade mPIN lesions in nearly 100% of the tested cohort by the age of 10 months [55]. To determine the effect of Pim1 overexpression after 10 months and to detect a possible cooperation of PIM1 overexpression and the loss of one *Pten* allele, we sacrificed WT, tgPim1, PTEN-Het and tgPim1/PTEN-Het mice at 10 months of age and screened the prostate for mPIN lesions. All but one of the WT mice failed to develop mPIN lesions, whereas 89% of the tgPim1 mice displayed mPIN I (pl-grade 2) lesions and 11% mPIN II (pl- grade 5) lesions. Sixty percent of the Pten-Het mice developed high grade prostate hyperplasia mPIN II–IV and 20% developed microinvasive carcinoma. TgPim1 expression in the PTEN-Het background showed prostate neoplasias similar to Pten-het alone (Figure 3). Sixty-six percent of the tgPim1/Pten-Het mice displayed mPIN II–IV lesions but no carcinomas (Fig. 2D).

### Overexpression of the Pim1 transgene leads to an impaired immune response in hormone-treated mice

**Figure 4:**
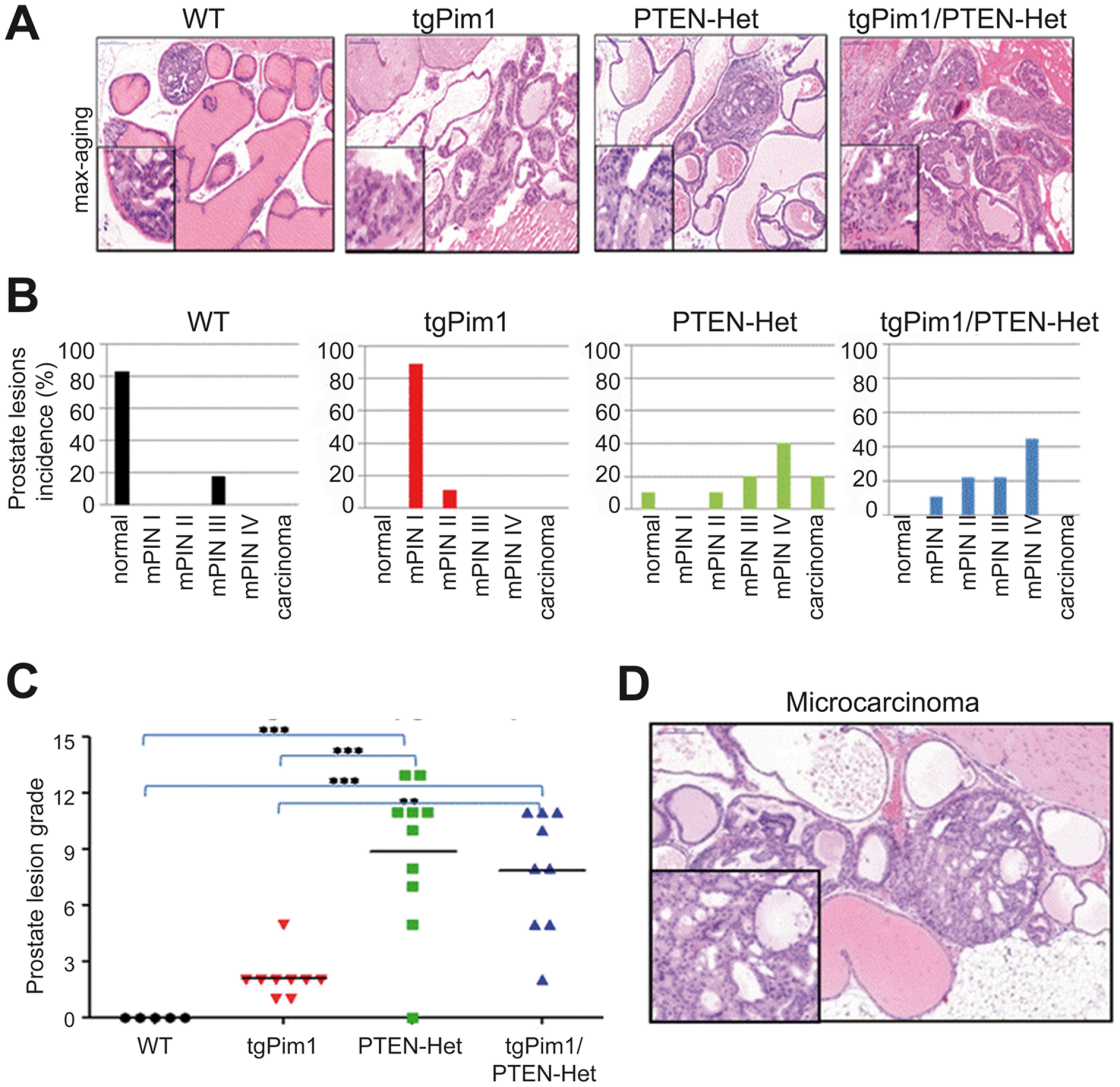
mPIN lesions in 10-month-old untreated mice. To determine the development of mPIN lesions over time without hormone treatment, 10-month-old untreated mice of each genotype were sacrificed and prostate tissue was obtained. H&E staining of prostate tissue was used for mPIN grading, applying the Bar Harbor grading system, and subdivided into mPIN I-IV as described in Table S6. **A**) Representative picture of the maximum grade reached: wt) grade 6; tgPim1) grade 5; PTEN-Het) grade 13; tgPim1/PTEN-Het) grade 11. **B**) Incidence (in %) of mPIN lesions per genotype in 10-month-old mice. Percentage of developed mPIN grade (mPIN I-IV and microinvasive carcinoma) was determined for each genotype using H&E staining of prostate tissue. **C**) mPIN lesions in 10-month-old untreated mice. The graphs represent the grading observed in the different prostates analyzed; statistical relevance is also shown: * = <0.05; ** = <0.01; *** = <0.001. **D:** H&E staining of a microcarcinoma. Microcarcinoma in a 10-month-old PTEN-Het mouse.

Hormone treatment with testosterone and estradiol (ratio 10∶1) induces low levels of prostate inflammation in treated animals, primarily as a result of estradiol. Animals with a normal immune response seem to be largely unaffected by hormone treatment, as high levels of inflammation have not been reported. However, in this study, tgPim1/PTEN-Het mice showed a significantly increased inflammation rate (55%) during the first round of hormone treatment, compared to all other genotypes (see figure 3B). Although TgPim1 mice did show slightly increased inflammation levels during the first round of treatment (20%), they displayed significantly increased inflammation during the 2nd round of treatment (66%, Fig. 4A and B). The analysis of the levels of IL-6 present in the stroma, as a marker for inflammation, showed a clear relation of the presence of this cytokine with the inflammation observed in the prostate (Figure S3). Due to this increased inflammatory response, 50% of the tgPim1/Pten-Het developed pyelonephritis during the first round of treatment, and 45% of the tgPim1 mice did so during the second round (figure 3 C and D). Because pyelonephritis is quite painful and leads to death within 12–24 hours, the animals were sacrificed at the earliest sign of kidney problems; no further rounds of hormone treatment were performed due to the high rate of affected animals. In our experiments we observed a clear relationship between the expression of PIM1 transgene and the development of pyelonephritis but appears only in the presence of hormone treatment (Table 1).

**Table 1:** Number of mice in which pyelonephritis was observed among all mice analyzed.

	Genotype			
Hormone Treatment	Wt	tgPim1	tgPim1/PTEN het	PTEN het
no treatment	0/9	0/12	0/6	0/6
1st round	0/17	8/12	8/17	0/16
2nd round	0/25	18/23		
3rd round	0/12			

Mice were subject to none, one, two or three rounds of hormone treatment as described in M&M, and pyelonephrytis analyzed. Table shows the number of mice with pyelonephritis/total mice analyzed for each genotype.

### Senescence as a barrier for progression to prostate carcinoma

**Figure 5:**
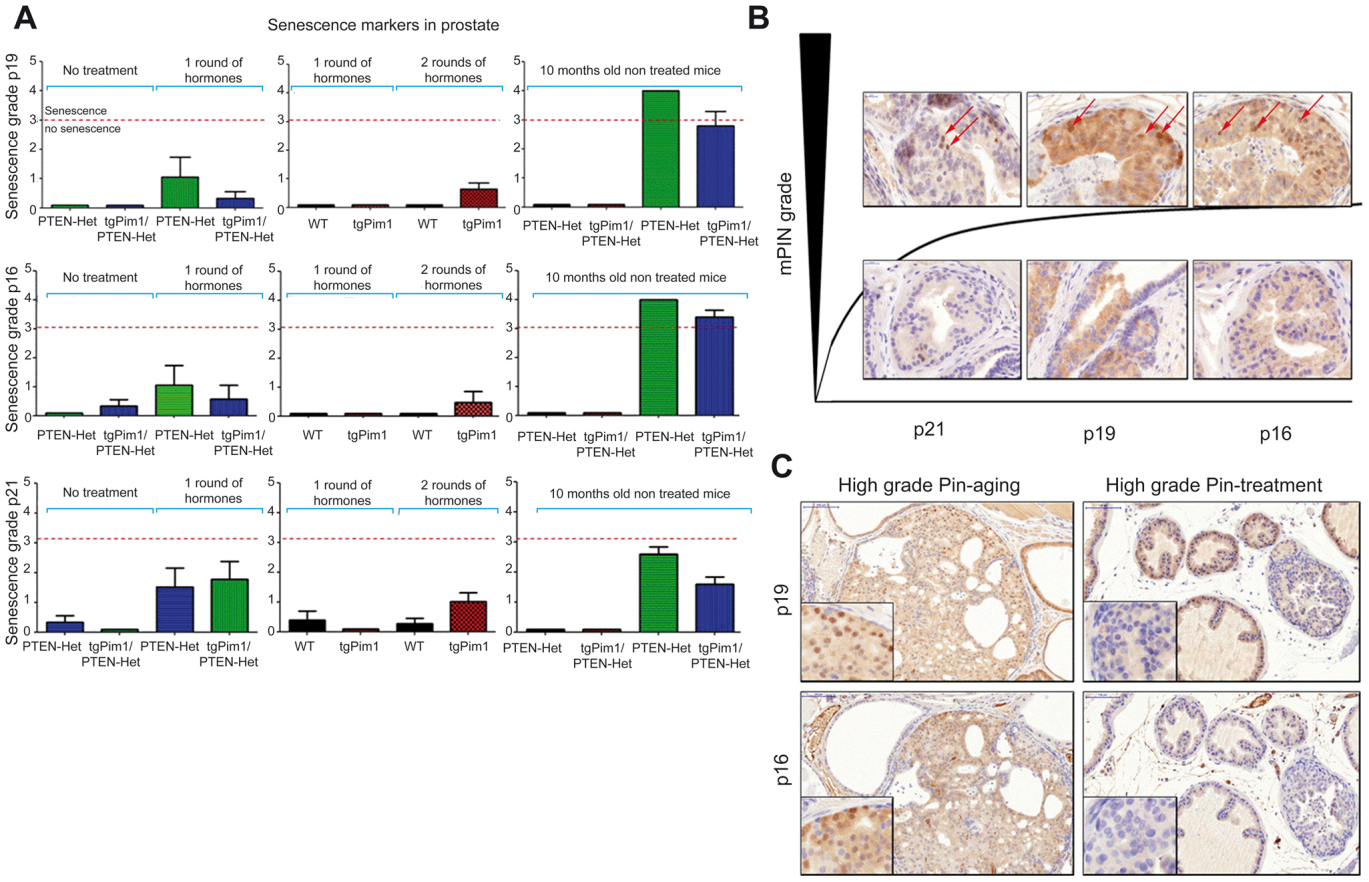
Senescence in prostate lesions. To determine senescence in prostate lesions, immunohistochemistry for several senescence markers, such as p21, p16 and p19, was performed in the prostate tissues of mice showing high grade and low grade mPIN lesions (10-month-old untreated mice and 16-week-old hormone treated mice). **A**) Senescence markers in prostate lesions. To determine senescence in prostate lesions from 16-week-old mice before and after hormone treatment, as well in 10-month-old untreated mice, immunohistochemistry for several senescence markers, namely p21, p16 and p19, was performed on the prostate tissues of mice of each genotype using the following grading scale for senescence grade (s-grade): s-grade 1 - few cells in 1 lesion (1–5% positive cells); s-grade 2 - few cells in more than one lesion; s-grade 3 - several cells (5–20%) in more than one lesion; s-grade 4 - more than 20% positive cells in more than one lesion; only concluding true senescence if s-grades 3 or 4 were reached in the same animal for at least 2 senescence markers preferably p16 and p19. **B**) Representative picture showing that senescence markers appear only in high grade mPIN in untreated mice. **C**) Senescence markers in high grade lesions – aging vs. hormone treatment. Immunohistochemistry for p16 and p19 in high grade mPIN lesions (mPINIV) in a 10-month-old untreated PTEN-Het mouse and a 16-week-old hormone treated PTEN-het mouse.

The process of senescence increases the levels of senescence markers, such as p21waf1, p16ink4a and p19ARF, in the cell nucleus; these markers can be detected by immunohistochemistry. Because few of the 16-week-old hormone-treated mice and 10-month-old untreated mice displayed high grade mPIN lesions or microinvasive carcinoma, we sought to determine senescence levels in the prostate tissues of mice of each genotype using the markers p21, p16 and p19 (Fig. 5). To quantify senescence, we used the following grading scale for the number of cells showing senescence markers: s-grade 1 - few cells in 1 lesion (1–5% positive cells); s-grade 2 - few cells in more than one lesion; s-grade 3 - several cells (5–20%) in more than one lesion; and s-grade 4 - more than 20% positive cells in more than one lesion. We considered a lesion of have true senescence only if s-grade 3 or s-grade 4 was reached for at least 2 of the 3 senescence markers in the same lesion.

The quantification of all three markers for all lesion grades in all cohorts showed that senescence only appeared in high grade lesions (Fig. 5) of 10-month-old untreated mice (Pten-Het and tgPim1/Pten-Het genotypes). We did not observe a high number of cells showing nuclear staining for p16, p19 nor p21 in low grade mPIN in any cohort. Furthermore, although hormone-treated mice displayed high grade lesions, we did not detect senescence markers in these mPIN IV lesions or in microinvasive carcinomas (Fig. 5, Figure S2).

To validate the arrest of senescence, we stained these lesions with a Ki67 marker for proliferation (Table 2). We observed different cell behaviors in hormone-treated vs. untreated animals. In treated animals, only high grade lesions showed immunostaining for Ki67, but no signs of senescence were observed. In untreated animals, we did not observe Ki67 immunostaining in low grade lesions from the WT or tgPim1 mice, and high grade lesions were not observed in these animals. However, we observed Ki67 staining in low grade lesions of Pten-Het mice, indicating some proliferative capability. Furthermore, high grade mPIN showed little or no Ki67 immunostaining indicating proliferative arrest (Table 2).

**Table 2:** KI67 levels in mPIN lesions.

Genotype	Grade	Hormone Treatment	Aging (10 months)
**WT**	**Low**	-	-
	**High**	NL	NL
**TgPim1**	**Low**	-	-
	**High**	+++	NL
**PTEN-Het**	**Low**	-	++
	**High**	+++	-
**TgPim1;PTEN-Het**	**Low**	-	NA
	**High**	++	+

Low grade: mPIN I and mPIN II. High grade: mPIN III and mPIN IV. In yellow conditions in which senescence markers were observed. NL: No lesions observed; NA: Not assessed.

## Discussion

Pim1 has been implicated in prostate cancer as a prognostic factor [56], [57]. Recent studies have also correlated Pim1 kinase with chemoresistance in prostate cancer cells, which is a common occurrence in more aggressive, hormone-refractory prostate cancers [52], [53]. However, we found overexpression of Pim1 only has a weak oncogenic effect in the prostate, as previously described in lymphoma. At 10 months of age, only low grade mPIN was observed.

The hormone treatment induced more frequent mPIN lesions and lesions of a higher grade (up to carcinoma over the course of 2 treatment cycles) in tgPim1 mice, compared to WT mice, with both genotypes beginning with lesion-free prostates. This finding indicates that the sole overexpression of Pim1 in the prostate is sufficient to increase hormone treatment-induced mPIN formation. Mice with one *Pten* allele inactivated (Pten-Het mice) and mice overexpressing Pim1 and having only one *Pten* allele (tgPim1/Pten-Het mice) showed low grade mPIN lesions before hormone treatment, and had a significantly increased incidence and percentage of high grade lesions after one round of hormone treatment. The fact that one treatment cycle was sufficient to induce high grade mPIN lesions in Pten-Het mice demonstrates the malignant potential of *Pten* loss, even though no carcinoma was detected. Furthermore, although there were no significant differences in mPIN grade severity in Pten-Het and tgPim1/Pten-Het mice, there was a trend of increased severity indicating cooperation between *Pten* loss and *PIM1* overexpression in hormone- induced mPIN.

Similar to other transgenic or KO models in the prostate [58], [59], [60], [61], our model showed that increased expression of Pim1 alone, or in combination with loss of one Pten allele, was not sufficient to produce adenocarcinoma; however, Pim expression clearly contributed oncogenically to the increased severity of mPIN, similar to other models reported. This finding is consistent with reports on prostate cell lines that showed Pim1 overexpression alone was not sufficient to malignantly transform benign cells but did enhance the *in vitro* and *in vivo* tumorigenic capabilities of tumor cells [48], [49]. Similarly, mice expressing Pim1 in T-cells were more susceptible to carcinogenesis-induced T-cell lymphoma [17], [62]. It has also been reported that transgenic mice expressing human Pim3 selectively in the liver have increased frequency and decreased latency of hepatocellular carcinoma induced by the carcinogen diethylnitrosamine [63]. Combined with our data, these studies suggest that while Pim family members are weak oncogenes, they can contribute to tumorigenesis by selectively enhancing tumorigenic capabilities related to progression.

The extent of the PIM-induced effects seems to depend on the affected tissues and on the natures of the pathways that are activated by the cooperating oncogenes. PIM1 is weakly oncogenic in naive adult mouse prostatic epithelium, however, it synergizes with c-MYC to induce prostate cancer within 6-weeks [64]. Importantly, c-MYC/Pim1 tumors seemed not to be dependent of c-MYC activity by S62 phosphorylation. It is understood that the mere overexpression of MYC initiates an apoptotic response that must be overcome to allow tumorigenesis [19], [39]. PIM kinases (PIM1 and PIM2) have been shown to counteract MYC-induced apoptosis through the phosphorylation of BAD, through decreases in cellular proapoptotic responses, and through increases in the protein stability and transcriptional activity of MYC [48], [51], [65]. This hypothesis could explain the results of Wang and colleagues [66] showing that Pim1 depletion by RNA interference in mouse and human prostate cancer cells decreased cellular proliferation, survival, Erk signaling and tumorigenicity even when MYC levels were not significantly altered. These results indicate that PIM1 may be necessary to maintain tumorigenicity in tumors with deregulated MYC. This phenomenon has also been shown in human prostate cancer, in which PIM1 is most likely to collaborate with MYC in cellular transformation as it is the most consistently expressed gene among MYC-positive and MYC-negative prostate cancer tumor samples [64], [65]. Furthermore, in human prostate tumors, coexpression of c-MYC and PIM1 is associated with higher Gleason grades [64].

Androgen receptor, AR, turnover has been previously shown to be critical for the proliferation of prostate cancer cells [67], [68], [69]. PIM1 kinase may regulate AR stability and translational activity in a phosphorylation-dependent manner [70]. We have observed that neither PIM1 transgene expression nor PTEN loss of one allele significantly increased AR expression in prostate (Table S7), however, some increase in AR nuclear levels can be observed in mice overexpressing PIM1 that were previously treated with hormones. It could be that the hormones are increasing PIM levels by stabilizing this protein, or PIM1 itself stimulate growth in a hormone-dependent fashion. In accordance with this last hypothesis it has been published that PIM1-dependent phosphorylation of AR impacts in gene transcription and is prevalent in aggressive prostate cancer [71], [72].

### Pim and inflammation

Hormone treatment is known to induce mPIN lesions and prostate adenocarcinoma in part through prostate inflammation, as prostatitis is associated with increased mPIN grade, pointing to a probable initiator role for inflammation in the early steps of prostate cancer [73], [74]. In our study, the inflammatory response of WT and Pten-Het mice appeared normal. Although there were small areas of inflammation in up to 20% of the treated mice, the WT and Pten-Het mice displayed a normal immune response and did not develop abscesses or pyelonephritis. However, animals expressing the PIM1 transgene (tgPim1 and tgPim1/Pten-Het mice) seemed to develop an increased immune response. This was confirmed by the stromal levels of IL-6, which were increased only in tissues with inflammation regardless of their genotype (Figure S3).

Normal prostate tissue contains stromal intraepithelial T- and B-lymphocytes [75], [76], macrophages and mast cells. In prostate inflammation, Th1 responses (IFN-γ, TNFα) and Th2 responses (IL-4, IL-5, IL-13) are activated, in addition to the expression of IL-6, IL-8 and IL-10, and NFκB activation [77]. Because IL-6, NFκB and Stat3 increase endogenous Pim1 expression, there would be an additional increase of Pim1 available in prostate tissues due to this positive feedback loop, possibly explaining an impaired immune response; Pim1 has been implicated in inflammation in several *in vitro* and *in vivo* models [78], [79]. Pim1 and Pim2 have also been shown to belong to an endogenous pathway that regulates T-cell growth and survival [80], [81]. Pim1 has been shown to regulate human Th1 cell differentiation [82] and play a role in immune cell activation and proliferation [41]. In addition, Pim1 appears to contribute to NFκB activation upon TNF-α activation [83] via a feedback loop, while Pim1-inhibitors prevent NFκB activation and decrease iNOS production in macrophages and decrease levels of TNFα.

Therefore, we speculate that the increased inflammation observed in PIM1 transgenic mice is due to the positive feedback loop between hormone treatment and PIM1 Tg expression. This hypothesis is indirectly supported by the fact that only mice expressing the PIM1 transgene and were subjected to hormone treatment developed inflammation and subsequent pyelonephritis (table 2). Mechanistically, it is possible that in this context, high PIM1 levels increased the recruitment of tumor/inflammation associated macrophages, MDSCs, mast cells and neutrophils to the target tissue, which can increase locally IL6 and other cytokines (such as IL1 and TNFa) and chemokines (such as CCL2 and CXCL8). It is possible that the increase in these cytokines in the extracellular media surrounding tumor cells might promote tumorigenesis by activating NFKb and/or STAT3 pathways [77].

### Effects of PIM1 expression on the prostate at 10 months of age

At 10 months of age, the prostates of WT mice showed no lesions, while Pten-het mice displayed high grade hyperplasia [84] (Fig. 3). Overexpression of Pim1 in the prostate slightly increased mPIN lesion grade, but only low grade lesions were observed in the tgPim1 mice. Surprisingly, Pim1 overexpression and Pten loss in the untreated animals did not seem to cooperate, although 60% of the animals displayed high grade mPIN lesions, none progressed to carcinoma. This finding might be explained by the senescence observed in high grade lesions in untreated 10-month-old animals.

We did not detect senescence in the low grade mPIN lesions of untreated 16-week-old mice or in hormone-treated animals of any genotype (1^st^ and 2^nd^ treatment round), even in severe high grade lesions. Significantly higher levels of senescence were detected in the high grade mPIN lesions of 10-month-old Pten-Het and tgPim1/Pten-Het mice, but not in tgPim1 or WT mice; senescence seems to be restricted to high grade lesions.

The mechanism of senescence in an inflammatory environment is not yet fully clear. Despite progressing to high grade mPIN, hormone-induced hyperplasias did not show senescence markers (we would like to remark that we were unable to detect SA-β-gal staining in mouse tissue, most likely due to technical problems). These high grade mPIN lesions correlated with samples demonstrating inflammation. Inflammation has been proposed to abolish cyclin-dependent kinase inhibitors expression during senescence, thus facilitating tumorigenesis [85], [86], [87]. Therefore, it is possible that in the high grade mPIN lesions observed during treatment the markers p16ink4a, p19ARF and p21waf1 are repressed by inflammation, possibly contributing to tumorigenesis. The data from KI67 seem to support this hypothesis.

There are many different hypotheses explaining the role of senescence in tumorigenesis. The most prevalent is that benign hyperplasias are mostly the result of the hyperproliferation of cells subject to oncogenic stress, which in the presence of immortalizing events (i.e., p53 mutations) can reach malignant status [88], [89]. Pandolfi and collaborators [90] proposed that *Pten*-loss pre-neoplasic lesions in the prostate are a consequence of oncogenic-stress activation and that lesions do not progress to malignant grades due to the activation of a senescent program that is independent of DNA-damage signaling and does not require previous hyperproliferation [90]. Our data in the prostate suggest that senescence markers appear only once the proliferative capability of oncogene-stressed cells is exhausted, after reaching high grade mPIN, explaining the lack of senescence markers in low grade mPIN. This finding also explains why we have not observed a linear correlation between mPIN and senescence grade.

While Pim1 is a weak oncogene [91] with little or no role in tumor initiation, it may play a role in tumor progression. The extent of the role of Pim1 in tumorigenicity seems to depend on the tissue affected and the nature of the molecular pathways activated by the co-expressing oncogenes; overexpression of cMyc and Pim1 in lymphoid tissues resulted in the death of mice in utero from pre-B cell lymphoma [91]. Our data also indicate that inflammation abolishes the senescent response, thus allowing high grade lesions to maintain their proliferative capacity and contribute to cancer.

## Supporting Information

Table S1 **Primers used for genotyping of transgenic mouse.**
(DOC)

Table S2 **Total dose of implanted hormones.**
(DOC)

Table S3 **Primers and protocols for Pim cDNA amplification.**
(DOC)

Table S4 **Used primary antibodies for immunohistochemistry and Western Blot.**
(DOC)

Table S5 **Used secondary antibodies for immunohistochemistry and Western Blot.**
(DOC)

Table S6 **Characterization and classification of mPIN.** All data from Ref. Shappell/and Park2002. “*mPin is the neoplastic proliferation of epithelial cells within preexisiting or normal basement membrane confined gland spaces with or without documented progression to invasive carcinoma. These epithelial cells demonstrate nuclear atypia and stratification. The foci can aquire a tufting, microcapillary, or cribiform growth.*” Shappell et al, Bar Harbor meeting report; Cancer Research 64, 2004.
(DOC)

Table S7 **Levels of Androgen Receptor staining in prostate tissue.** AR levels were visually assessed by 2 independent observations. Levels were evaluated according to nuclear intensity.
(DOC)

Table S8 **Number of mice analyzed in the current work.** The prostate of each of these mice was processed and stained for H&E, senescence markers (p16, p21 and p19) and visually analyzed at the microscopy.
(DOC)

Figure S1 **Grading example for prostate intraepithelial neoplasia (mPIN) in genetically engineered mice.** 16 week old male mice were sacrificed and prostate tissue was taken and prepared for immunohistochemistry. H&E staining of prostate tissue was used for mPIN grading. mPIN grades were transferred to a numeric system subdividing each mPIN grade into subgrades depending on the quantity of affected glands. Subgrades are: focal, multifocal (more than 3 glands of 1 lobe affected) or diffuse (more than 30% of 1 lobe affected) as explained above.
(DOC)

Figure S2 **Differentiation of mPIN IV grade lesions and carcinoma 10 month old untreated mice.** H&E staining of prostate tissue of 10 month old mice was used for mPIN grading. Cytokeratin 14 and smooth muscle actin staining were done to differentiate mPIN lesions (as in tgPim1/PTEN-Het mice) from microinvasive carcinoma in (as in PTEN-Het mice). Red arrows indicate intact SMA staining- mPN IV lesion, black arrows indicate were SMA staining was negative – carcinoma.
(DOC)

Figure S3 **Levels of IL-6 correlated with inflammation.** 16 week old male mice were sacrificed and prostate tissue was taken and prepared for immunohistochemistry to detect extracellular IL-6. These data was correlated to inflammation levels. The panels are as indicated: **A,C,E,G** no inflammation; **B,D,F,H** inflammation. The genotypes are as follows: **A,B**: wt; **C,D**: tg PIM1; **E,F**: PTEN-Het; **G, H**: tg PIM1/PTEN-Het.
(DOC)
