# Correction: Conditional Transgenic Expression of PIM1 Kinase in Prostate Induces Inflammation-Dependent Neoplasia

**DOI:** 10.1371/annotation/1b8b6002-4e0f-4c5d-8ae8-fd45447b6149

**Published:** 2013-05-09

**Authors:** Maja Narlik-Grassow, Carmen Blanco-Aparicio, Yolanda Cecilia, Marco Perez, Sandra Muñoz-Galvan, Marta Cañamero, Oliver Renner, Amancio Carnero

Oliver Renner was omitted from the author byline.

He should be listed as the seventh author and affiliated with: Experimental Therapeutics programme, Spanish National Cancer Research Centre, Madrid, Spain

The contributions of this author are as follows: Conceived and designed the experiments, Performed the experiments, Analyzed the data. 

