# Supplementary material for: Conditional Transgenic Expression of PIM1 Kinase in Prostate Induces Inflammation-Dependent Neoplasia
Source: PLoS One. 2013 Apr 2;8(4):e60277. doi: 10.1371/journal.pone.0060277 (PMC3614961; doi:10.1371/journal.pone.0060277)
Supplement: Table S1 — Primers used for genotyping of transgenic mouse. (DOC) [file pone.0060277.s001.doc]

**Table S1: Primers used for genotyping of transgenic mouse lines: Primer sequences and PCR programmes were specifically designed for genotyping of mice. PCR fragments were seperated on a 1,5% agarose gel..**

| **Gene** | **Primer sequence (5´- 3´)** | **Thermocycler programme** | **Expected band (bp)** | | | |
| --- | --- | --- | --- | --- | --- | --- |
|  |  |  | **(+/+)** | **(+/-)** | **(-/-)** | **(-/T)** |
| *Pim1* | Fw: CGAGATCGCCATATTTGGTGTCCCCGAG | 1. 95°C 5 min 2. 95°C 45 sec 3. 65°C 30 sec 4. 72°C 45 sec to # 2 x 34 cycl. 5. 72°C 5 min 6. 4°C pause |  |  |  | 350 |
|  | Rev: CCAGCTTGGTGGCGTGCAGGTCGTTGCA |  |  |  |  |  |
| *pten* | Fw: TCATCTTCACTTAGCCATTG | 1. 94°C 4 min 2. 94°C 1 min 3. 57°C 1 min 4. 72°C 2 min to # 2 x 39 cycl. 5. 72°C 10 min 6. 4°C pause | 900 | 900/ 1000 | 1000 |  |
|  | Rev: ACTCAAGGCAGGGATGAG |  |  |  |  |  |
| *Psa61* | Fw: CTTGTAGGGTGACCAGAGCAG | 1. 95°C 5 min 2. 95°C 1 min 3. 59°C 1 min 4. 72°C 1 min to # 2 x 34 cycl. 5. 72°C 7 min 6. 4°C pause |  |  |  | 330 |
|  | Rev: GCAGGCATCCTTGCAAGATG |  |  |  |  |  |
