# Supplementary material for: Conditional Transgenic Expression of PIM1 Kinase in Prostate Induces Inflammation-Dependent Neoplasia
Source: PLoS One. 2013 Apr 2;8(4):e60277. doi: 10.1371/journal.pone.0060277 (PMC3614961; doi:10.1371/journal.pone.0060277)
Supplement: Table S2 — Total dose of implanted hormones. (DOC) [file pone.0060277.s002.doc]

Table S2: Total dose of implanted hormones

|  | Implanted total dose  at 8 weeks of age | Implanted total dose at 16 weeks of age |
| --- | --- | --- |
| testosterone | 12,5 mg | 18,75 mg |
| β-Estradiol | 1,25 mg | 1,87 mg |
