# Supplementary material for: Conditional Transgenic Expression of PIM1 Kinase in Prostate Induces Inflammation-Dependent Neoplasia
Source: PLoS One. 2013 Apr 2;8(4):e60277. doi: 10.1371/journal.pone.0060277 (PMC3614961; doi:10.1371/journal.pone.0060277)
Supplement: Table S3 — Primers and protocols for Pim cDNA amplification. (DOC) [file pone.0060277.s003.doc]

**Table S3: Primers and protocols for Pim cDNA amplification.** Specifically designed primers were used to amplify cDNA obtained through reverse transcription-PCR after total RNA extraction from tissues. Fragment length was controlled on 1.5% agarose gels.

| **Gene** | **Primer sequence (5´- 3´)** | **Thermocycler programme** | **Expected band (bp)** | | | |
| --- | --- | --- | --- | --- | --- | --- |
|  |  |  | **(+/+)** | **(+/-)** | **(-/-)** | **(+/T)** |
| *Pim1* | Fw: CAAGGACGAAAACATCCTTATC | 1. 95°C 5 min 2. 95°C 45 sec 3. 66°C 30 sec 4. 72°C 45 sec to # 2 x 37 cycl. 5. 72°C 5 min 6. 4°C pause |  |  |  | 500 |
|  | Rev: GATGGGACCCGAGTGTATAGCC |  |  |  |  |  |
| *gapdh* | Fw: AAGGTCGGTGTGAACGGATT | 1. 95°C 5 min 2. 95°C 1 min 3. 59°C 1 min 4. 72°C 1 min to # 2 x 34 cycl. 5. 72°C 7 min 6. 4°C pause | 1000 |  |  |  |
|  | Rev: TTGCTGGGGTGGGTGGTC |  |  |  |  |  |
