# Supplementary material for: Conditional Transgenic Expression of PIM1 Kinase in Prostate Induces Inflammation-Dependent Neoplasia
Source: PLoS One. 2013 Apr 2;8(4):e60277. doi: 10.1371/journal.pone.0060277 (PMC3614961; doi:10.1371/journal.pone.0060277)
Supplement: Table S4 — Used primary antibodies for immunohistochemistry and Western Blot. (DOC) [file pone.0060277.s004.doc]

Table S4: Used primary antibodies for immunohistochemistry and Western Blot.

| **Primary Antibody** | **Manufacturer** | **Cat. number** | **IHQ** |
| --- | --- | --- | --- |
| p21 | Santa Cruz | sc-397-G | 1 : 300 |
| p19^ARF^ | Santa Cruz | sc-32748 | 1 : 50 |
| p16 | Santa Cruz | sc-1207 | 1 : 50 |
| AR | Santa Cruz | sc-816 | 1 : 50 |
| Cytokeratin 14 | Thermo Scientific | RB-9020-P0 | 1 : 4500 |
| Smooth muscle actin | Thermo Scientific | 090309F | 1 : 250 |
