# Supplementary material for: Conditional Transgenic Expression of PIM1 Kinase in Prostate Induces Inflammation-Dependent Neoplasia
Source: PLoS One. 2013 Apr 2;8(4):e60277. doi: 10.1371/journal.pone.0060277 (PMC3614961; doi:10.1371/journal.pone.0060277)
Supplement: Table S5 — Used secondary antibodies for immunohistochemistry and Western Blot. (DOC) [file pone.0060277.s005.doc]

Table S5: Used secondary antibodies for immunohistochemistry and Western Blot.

| **Secondary Antibody** | **Manufacturer** | **Cat. number** | **WB** | **IHQ** |
| --- | --- | --- | --- | --- |
| Omnimap (anti-rabbit) | Ventana | 760-4311 |  | Predil. |
| Horse raddish peroxidase (goat anti-rabbit) | Dako | P0448 |  | 1 : 50 |
| Biotinylated-IgG (rabbit anti-goat) | Dako | E0446 |  | 1 : 200 |
| Rat-HRP on mouse | Biocare | RT517L |  | Predil. |
| IgG (rabbit anti-mouse) | Epitomics | 3024-I |  | 1 : 500 |
