# Supplementary material for: Conditional Transgenic Expression of PIM1 Kinase in Prostate Induces Inflammation-Dependent Neoplasia
Source: PLoS One. 2013 Apr 2;8(4):e60277. doi: 10.1371/journal.pone.0060277 (PMC3614961; doi:10.1371/journal.pone.0060277)
Supplement: Table S6 — Characterization and classification of mPIN. All data from Ref. Shappell/and Park2002. “mPin is the neoplastic proliferation of epithelial cells within preexisiting or normal basement membrane confined gland spaces with or without documented progression to invasive carcinoma. These epithelial cells demonstrate nuclear atypia and stratification. The foci can aquire a tufting, microcapillary, or cribiform growth.” Shappell et al, Bar Harbor meeting report; Cancer Research 64, 2004. (DOC) [file pone.0060277.s006.doc]

Table S6: Characterization and classification of mPIN. **All data from Ref. Shappell /and Park2002.** *"mPin is the neoplastic proliferation of epithelial cells within preexisiting or normal basement membrane confined gland spaces with or without documented progression to invasive carcinoma. These epithelial cells demonstrate nuclear atypia and stratification. The foci can aquire a tufting, microcapillary, or cribiform growth."* Shappell et al, Bar Harbor meeting report; Cancer Research 64, 2004.

| **mPIN grade** | **prostate lesion grade (pl-grade) / localization** | **Characteristics** |
| --- | --- | --- |
| normal | 0 | 1 cell layer of normal cells |
| mPIN I | 1/focal 2/multifocal 3/diffuse | small foci, 2 layers of atypical cells, stroma intact, cells larger and taller than normal cells |
| mPIN II | 4/focal 5/multifocal 6/diffuse | > 2 layers of atypical cells, stroma intact, increasing but not severe nuclear pleomorphism and hyperchromasia, cells larger and taller than normal cells, larger nuclei and vesicular chromatin pattern |
| mPIN III | 7/focal 8/multifocal 9/diffuse | foci almost fill lumen of ducts, stroma intact, gland outline smooth, atypical cells poorly orientated, increasingly severe nuclear pleomorphism and hyperchromasia, mitosis present |
| mPIN IV | 10/focal 11/multifocal 12/diffuse | foci fill lumen of ducts, duct profiles are distorted and irregular, stroma irregular or absent, epithelium still surrounded by laminin layer, lesions exted along duct to involve adjacent ducts, increased severe pleomorphism and hypercromasia |
| carcinoma | 13 | foci fill lumen of ducts, adjacent ducts involved, epithelium loses surrounding laminin layer |
