# Supplementary material for: Conditional Transgenic Expression of PIM1 Kinase in Prostate Induces Inflammation-Dependent Neoplasia
Source: PLoS One. 2013 Apr 2;8(4):e60277. doi: 10.1371/journal.pone.0060277 (PMC3614961; doi:10.1371/journal.pone.0060277)
Supplement: Table S7 — Levels of Androgen Receptor staining in prostate tissue. AR levels were visually assessed by 2 independent observations. Levels were evaluated according to nuclear intensity. (DOC) [file pone.0060277.s007.doc]

***Table S7*: Levels of Androgen Receptor staining in prostate tissue**. AR levels were visually assessed by 2 independent observations. Levels were evaluated according to nuclear intensity.

| **Hormone treatment** | **WT** | **PIM1 tg** | **PTEN-Het** | **PIM1tg/PTEN-het** |
| --- | --- | --- | --- | --- |
| Untreated | ++ | ++ | ++ | ++ |
| Treated 1 round | NA | +++ | ++ | +++ |
| Treated 2 rounds | ++ | +++ | ND | ND |

Levels were assessed according to: 0= no staining; += low staining; ++= clear medium staining; +++=very strong staining. NA= not analyzed; ND= mice not analyzed due to pyelonephritis with only one round of treatment
