# Supplementary material for: Conditional Transgenic Expression of PIM1 Kinase in Prostate Induces Inflammation-Dependent Neoplasia
Source: PLoS One. 2013 Apr 2;8(4):e60277. doi: 10.1371/journal.pone.0060277 (PMC3614961; doi:10.1371/journal.pone.0060277)
Supplement: Table S8 — Number of mice analyzed in the current work. The prostate of each of these mice was processed and stained for H&E, senescence markers (p16, p21 and p19) and visually analyzed at the microscopy. (DOC) [file pone.0060277.s008.doc]

***Table S8*: Number of mice analyzed in the current work.** The prostate of each of these mice was processed and stained for H&E, senescence markers (p16, p21 and p19) and visually analyzed at the microscopy.

| **H&E and Senescence markers** | **wt** | **tgPim1** | **tgPim1/PTEN** | **PTEN** |
| --- | --- | --- | --- | --- |
| no treatment | 9 | 4 | 6 | 6 |
| 1st round | 9 | 4 | 17 | 16 |
| 2nd | 17 | 15 |  |  |
| 3rd | 12 |  |  |  |
| Aging (10 months no treatments) | 6 | 12 | 15 | 13 |
