# Supplementary material for: Conditional Transgenic Expression of PIM1 Kinase in Prostate Induces Inflammation-Dependent Neoplasia
Source: PLoS One. 2013 Apr 2;8(4):e60277. doi: 10.1371/journal.pone.0060277 (PMC3614961; doi:10.1371/journal.pone.0060277)
Supplement: Figure S1 — Grading example for prostate intraepithelial neoplasia (mPIN) in genetically engineered mice. 16 week old male mice were sacrificed and prostate tissue was taken and prepared for immunohistochemistry. H&E staining of prostate tissue was used for mPIN grading. mPIN grades were transferred to a numeric system subdividing each mPIN grade into subgrades depending on the quantity of affected glands. Subgrades are: focal, multifocal (more than 3 glands of 1 lobe affected) or diffuse (more than 30% of 1 lobe affected) as explained above. (DOC) [file pone.0060277.s009.doc]

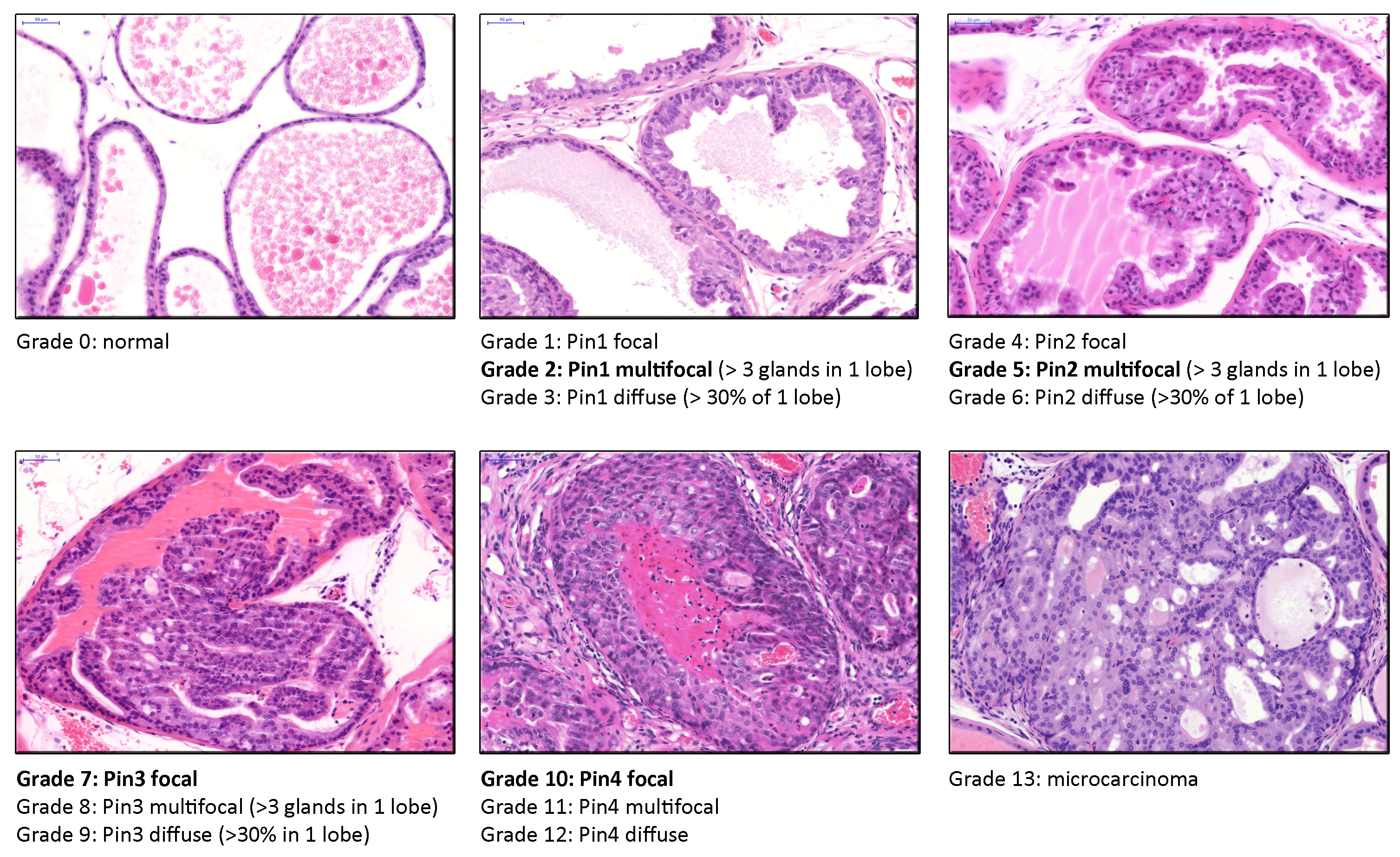


**Figure S1:** Grading example for prostate intraepithelial neoplasia (mPIN) in genetically engineered mice. 16 week old male mice were sacrificed and prostate tissue was taken and prepared for immunohistochemistry. H&E staining of prostate tissue was used for mPIN grading. mPIN grades were transferred to a numeric system subdividing each mPIN grade into subgrades depending on the quantity of affected glands. Subgrades are: focal, multifocal (more than 3 glands of 1 lobe affected) or diffuse (more than 30% of 1 lobe affected) as explained above.
