# Supplementary material for: Conditional Transgenic Expression of PIM1 Kinase in Prostate Induces Inflammation-Dependent Neoplasia
Source: PLoS One. 2013 Apr 2;8(4):e60277. doi: 10.1371/journal.pone.0060277 (PMC3614961; doi:10.1371/journal.pone.0060277)
Supplement: Figure S2 — Differentiation of mPIN IV grade lesions and carcinoma 10 month old untreated mice. H&E staining of prostate tissue of 10 month old mice was used for mPIN grading. Cytokeratin 14 and smooth muscle actin staining were done to differentiate mPIN lesions (as in tgPim1/PTEN-Het mice) from microinvasive carcinoma in (as in PTEN-Het mice). Red arrows indicate intact SMA staining- mPN IV lesion, black arrows indicate were SMA staining was negative – carcinoma. (DOC) [file pone.0060277.s010.doc]

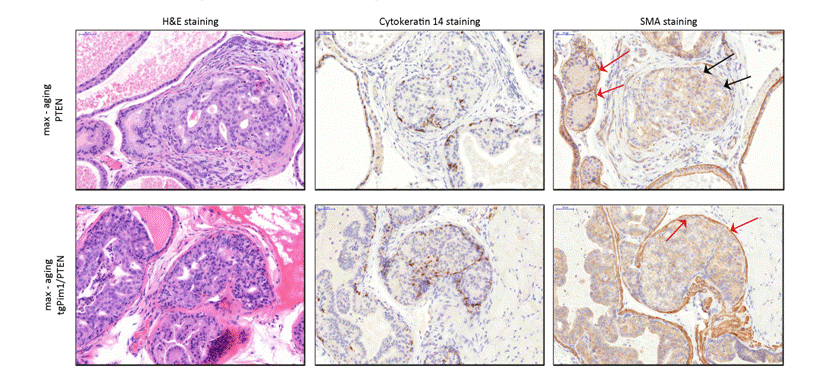


**Figure S2 :** **Differentiation of mPIN IV grade lesions and carcinoma 10 month old untreated mice**. H&E staining of prostate tissue of 10 month old mice was used for mPIN grading. Cytokeratin 14 and smooth muscle actin staining were done to differentiate mPIN lesions (as in tgPim1/PTEN-Het mice) from microinvasive carcinoma in (as in PTEN-Het mice). Red arrows indicate intact SMA staining- mPN IV lesion, black arrows indicate were SMA staining was negative – carcinoma.
