# Supplementary material for: Conditional Transgenic Expression of PIM1 Kinase in Prostate Induces Inflammation-Dependent Neoplasia
Source: PLoS One. 2013 Apr 2;8(4):e60277. doi: 10.1371/journal.pone.0060277 (PMC3614961; doi:10.1371/journal.pone.0060277)
Supplement: Figure S3 — Levels of IL-6 correlated with inflammation. 16 week old male mice were sacrificed and prostate tissue was taken and prepared for immunohistochemistry to detect extracellular IL-6. These data was correlated to inflammation levels. The panels are as indicated: A,C,E,G no inflammation; B,D,F,H inflammation. The genotypes are as follows: A,B: wt; C,D: tg PIM1; E,F: PTEN-Het; G, H: tg PIM1/PTEN-Het. (DOC) [file pone.0060277.s011.doc]

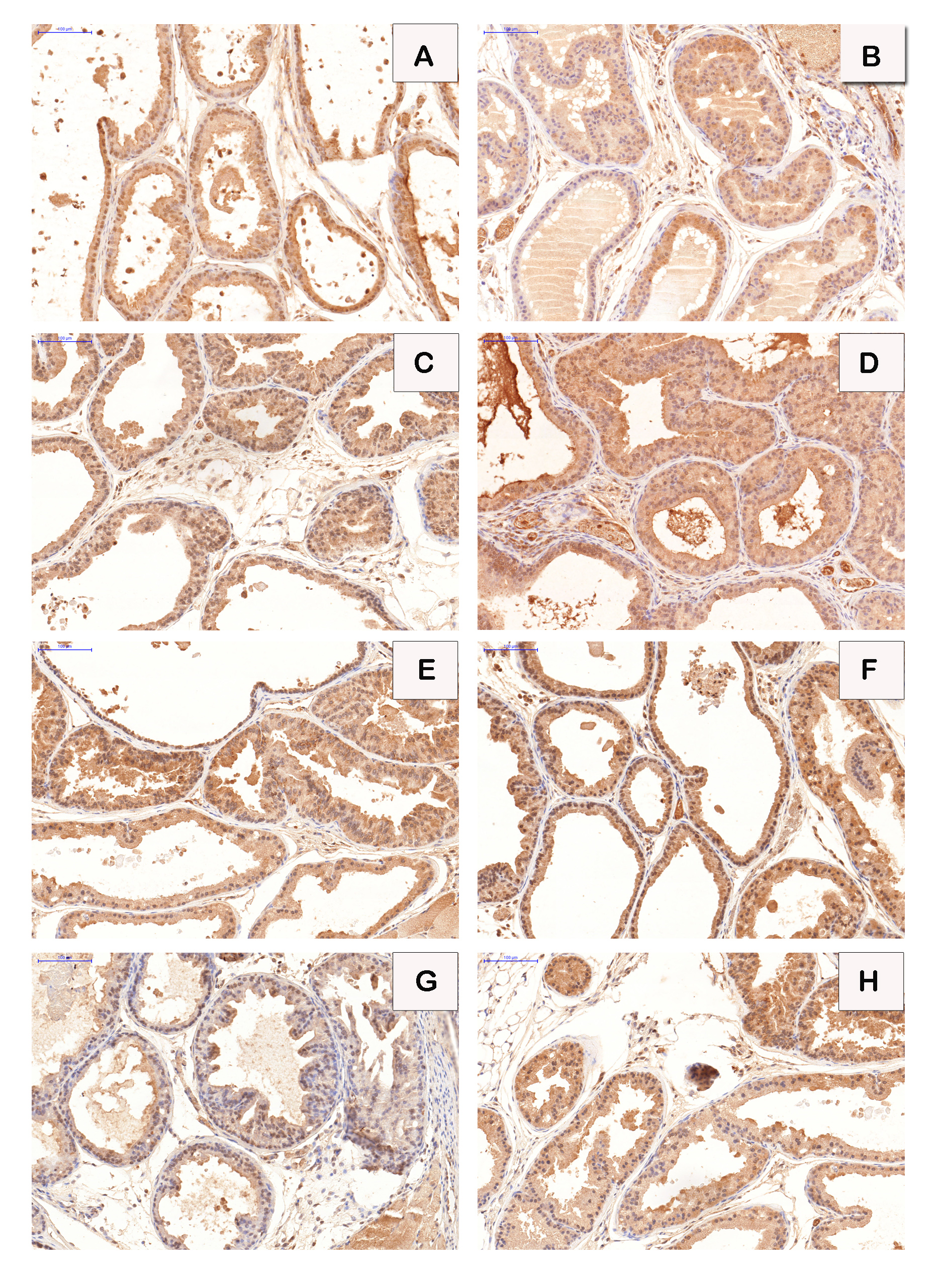


**Figure S3: Levels of IL-6 correlated with inflammation.** 16 week old male mice were sacrificed and prostate tissue was taken and prepared for immunohistochemistry to detect extracellular IL-6. These data was correlated to inflammation levels. The panels are as indicated: **A,C,E,G**  no inflammation; **B,D,F,H** inflammation. The genotypes are as follows: **A,B**: wt; **C,D**: tg PIM1; **E,F**: PTEN-Het; **G, H**: tg PIM1/PTEN-Het
